# Successful treatment of B-cell acute lymphoblastic leukemia with *SET-NUP214* rearrangement using venetoclax: A first case report and literature review

**DOI:** 10.1007/s00277-026-07190-y

**Published:** 2026-07-28

**Authors:** Gang Chen, Chao Min, Jing Xu, Jifu Zheng, Huihui Li, Zhenjiang Li

**Affiliations:** Department of Hematology, The Second Affiliated Hospital of Nanchang University, No.1 Minde Road, Donghu District, Nanchang, 330006 Jiangxi China

**Keywords:** B-cell acute lymphoblastic leukemia, *SET-NUP214*, Venetoclax, Chemoresistance, Allogeneic stem cell transplantation

## Abstract

B-cell acute lymphoblastic leukemia (B-ALL) harboring the *SET-NUP214* fusion constitutes an exceedingly rare and aggressive subtype. While this genetic aberration is a well-recognized hallmark of T-cell acute lymphoblastic leukemia (T-ALL), its clinical implications and therapeutic landscape in the B-cell lineage remain largely elusive. Herein, we report the first documented case of relapsed/refractory *SET-NUP214*-positive B-ALL achieving a successful response to a salvage regimen combining venetoclax with Hyper-CVAD chemotherapy.

A 25-year-old male presented with profound cytopenias and 95.5% bone marrow blasts. Flow cytometric analysis characterized the malignant population as pro-B-ALL, with immunophenotypic expression of CD34, CD38, HLA-DR, CD7, and CD58, alongside partial CD19 and cCD79a, and myeloid co-expression (CD13/CD33). Molecular profiling identified the *SET-NUP214* fusion transcript, accompanied by concurrent *JAK3*,* PHF6*,* and PTPN11* mutations. The patient experienced a short-term relapse following induction remission with the VDLP regimen, and subsequent re-induction with the VDEP regimen failed. Subsequently, MRD-negative remission was achieved after adding venetoclax during the mid-course of Hyper-CVAD-A chemotherapy. This deep response facilitated a successful bridge to allogeneic hematopoietic stem cell transplantation (allo-HSCT), with the patient maintaining sustained remission for 24 months post-transplant. Our findings suggest that venetoclax-based combinations may provide a potent reinduction strategy for refractory *SET-NUP214*-positive ALL, underscoring *BCL-2* inhibition as a pivotal therapeutic intervention for this high-risk genomic subset.

**Supplementary Information:**

The online version contains supplementary material available at https://doi.org/10.1007/s00277-026-07190-y.

## Key Points

First report of successful venetoclax use in *SET-NUP214* B-ALL.

Provides a new therapeutic option for this aggressive, treatment-resistant leukemia.

**Supplementary Information:**

The online version contains supplementary material available at https://doi.org/10.1007/s00277-026-07190-y.

## Introduction

Hematological malignancies are traditionally categorized by recurrent cytogenetic abnormalities. Among these, the *SET-NUP214* fusion gene—resulting from either a balanced translocation t(9;9)(q34;q34) or a cryptic interstitial deletion at chromosome 9q34—represents a rare but recurring aberration. First identified in 1992 by von Lindern et al. in a case of acute undifferentiated leukemia (AUL) [1], this fusion often eludes detection via conventional karyotyping due to the subtle nature of the cryptic del(9)(q34.11;q34.13) or t(9;9)(q34;q34). Consequently, fluorescence in situ hybridization (FISH) or reverse transcription-polymerase chain reaction (RT-PCR) remain the diagnostic gold standards. Although *SET-NUP214*-positive acute leukemia is relatively uncommon, it represents the second most frequent cytogenetic abnormality in T-cell acute lymphoblastic leukemia (T-ALL) [2]. Beyond the T-cell lineage, this fusion has been intermittently identified in acute myeloid leukemia (AML), AUL, mixed-phenotype acute leukemia (MPAL), and other lymphoid neoplasms [3–5]. Clinically, *SET-NUP214*-associated leukemias are characterized by a highly aggressive phenotype, marked by extensive extramedullary involvement, primary glucocorticoid resistance, and a dismal response to standard induction therapy, culminating in high relapse rates. Owing to its rarity, documented cases of B-cell acute lymphoblastic leukemia (B-ALL) harboring the *SET-NUP214* fusion remain sporadic, with a notable lack of large-scale cohort data. Here, we report a case of relapsed/refractory B-ALL with *SET-NUP214* fusion that achieved a deep second complete remission (CR2) following salvage therapy with venetoclax in combination with chemotherapy.

## Case report

In June 2023, a 25-year-old male was admitted to the Second Affiliated Hospital of Nanchang University (Jiangxi, China) presenting with a one-month history of progressive dizziness, fatigue, and generalized malaise. The patient’s family history was unremarkable for hereditary hematologic disorders. Physical examination revealed marked pallor of the skin and mucosal membranes, indicative of severe anemia. Initial laboratory evaluation demonstrated profound pancytopenia with paradoxical leukocytosis: hemoglobin 56 g/L, red blood cell (RBC) count 1.81 × 10¹²/L, white blood cell (WBC) count 57.86 × 10⁹/L (98.2% lymphocytes; absolute lymphocyte count 56.78 × 10⁹/L), and an absolute neutrophil count (ANC) of 0.70 × 10⁹/L. Peripheral blood smears identified 71% blasts. Bone marrow (BM) aspiration revealed hypercellularity with 95.5% blasts, predominantly comprised of medium-sized immature cells with round-to-irregular nuclei, fine chromatin, and inconspicuous nucleoli. Flow cytometric analysis characterized the malignant population as pro-B-ALL, with immunophenotypic expression of CD34, CD38, HLA-DR, CD7, and CD58, alongside partial CD19 and cCD79a, and myeloid co-expression (CD13/CD33). (See Supplementary Material 1 for details). BM biopsy demonstrated grade MF-1 reticulin fibrosis; immunohistochemical staining was positive for CD19, CD10, and CD34, with a BCL-2 expression rate of 70%. (Detailed pathological results are available in Supplementary Material 1). Cytogenetic analysis revealed a karyotype of 46,XY, del(11)(q22q24) [7]/46,XY [3]. Molecular profiling identified *SET-NUP214* fusion transcripts and a WT1/ABL transcript ratio of 28.6%. Furthermore, next-generation sequencing (NGS) detected pathogenic mutations in *JAK3*,* PHF6*,* JAK1*,* and PTPN11*. (Detailed results are provided in Supplementary Material 1).

Induction therapy with the VDLP regimen (vincristine, daunorubicin, L-asparaginase, and prednisone) commenced four days after admission. Although the patient initially exhibited corticosteroid resistance, complete remission (CR) was ultimately achieved. However, the induction phase was complicated by Serratia marcescens sepsis and a subsequent left plantar abscess, necessitated surgical debridement. This postoperative recovery delayed consolidation therapy by over one month. Two months later (August 2023), the patient experienced a rapid hematological relapse with 17% marrow blasts. Reinduction with the VDEP regimen (vincristine, daunorubicin, etoposide, and prednisone) failed to achieve disease control. In the fourth month after initial presentation (October 2023), the disease had progressed further, with 76% marrow blasts and the emergence of a palpable 1 × 0.8 cm mass in the right upper eyelid, indicative of extramedullary involvement. On October 7, 2023, the Hyper-CVAD-A regimen (cyclophosphamide, vincristine, doxorubicin, and dexamethasone) was initiated; despite an initial transient response—characterized by the clearance of peripheral blasts and regression of the eyelid lesion—the disease progressed on day 10, with blasts re-emerging in the peripheral blood (28%) and a recurrence of the eyelid mass. Faced with this refractory state, venetoclax was integrated into the treatment on day 12. One month later, the patient achieved a deep CR, with MRD negativity confirmed by both flow cytometry and real-time quantitative PCR. Following one subsequent cycle of Hyper-CVAD-B combined with venetoclax, the patient successfully underwent allogeneic hematopoietic stem cell transplantation (allo-HSCT) six months after presentation (December 2023). As of the submission of this manuscript, the patient remains in MRD-negative CR with only mild graft-versus-host disease (GVHD).

### Clinical characteristics of *SET-NUP214*-positive B-ALL: A literature synthesis

A systematic synthesis of the literature reveals that while *SET-NUP214* has been identified in molecular screening cohorts, only 10 cases of B-ALL (including MPAL with B-lineage involvement) have been documented with detailed clinicopathological data to date (Table 1). The present case represents the 11th documented instance with comprehensive clinical follow-up. Mirroring the clinicopathological hallmarks of its T-ALL counterpart, this entity exhibits a striking male predominance (90.9%; 10/11) and primarily affects young adults (median age: 29.4 years; range: 18–45). A defining feature is frequent lineage infidelity, characterized by the aberrant co-expression of CD7, CD13, and CD33 or CD3. The diagnostic spectrum is evenly bifurcated between classic B-ALL (45.5%) and MPAL (54.5%) with diverse lineage combinations. Genomic profiling underscores a complex landscape, with 27.3% of cases displaying complex karyotypes and recurrent co-mutations in *PHF6*, the *JAK-STAT* pathway (*JAK1/JAK3*), and *FLT3-ITD*. Historically, this subgroup is defined by profound therapeutic resistance, particularly to corticosteroids and intensive induction chemotherapy; notably, only one previously reported patient achieved a transient 5-month remission following a DA (daunorubicin and cytarabine) regimen. To the best of our knowledge, the present case represents the first documented instance of relapsed/refractory *SET-NUP214*-positive B-ALL achieving MRD-negative remission through the integration of venetoclax with Hyper-CVAD. This deep molecular response served as a successful bridge to allo-HSCT, ultimately resulting in sustained, long-term survival.

**Table 1:** Clinicopathological characteristics of SET-NUP214 positive acute leukemias with B-lineage involvement reported in the literature

Year	Diagnosis	Gender	Age, (years)	WBC count, x10^9 cells/l	Blasts, %	Immunophenotype	Diagnostic Methods	Karyotype	Gene abnormality	Treatment	RFS	OS	Outcome, months	Refs.
2010	B-ALL	M	42	-	-	-	-	Normal	*SET-NUP214*	CT	-	-	-	[6]
2014	MPAL B/M	M	19	217	93.5	CD7,CD11b, HLA-DR+,CD34+, CD38+, CD58+, CD79a+, CD19+ (dim), CD22+, CD33+, CD13+	Morphology, MFC, Karyotyping, FISH, RT-PCR	Complex	*FLT3-ITD*	CT	-	-	NR	[7]
2016	MPAL B/M	F	37	5.39	52	CD7,CD19,CD13,CD33,CD15,MPO, CD79a, HLA-DR+	Morphology, MFC, Karyotyping, FISH	46,XX, del(7),t(16;17)	-	CT	-	-	Relapsed, died	[8]
2020	MPAL B/T?	M	29	0.56	-	CD7, CD34, HLA-DR, CD10, CD19, CD33, CD117, cCD79a, cCD3	Morphology, MFC, Karyotyping, FISH	46,XY, add(6)t(?1;6),del (16)	-	CT+HSCT + CAR-T	-	-	Relapsed, alive + > 42 months	[9]
2021	B-ALL	M	18	217	93.5	HLA-DR+, CD34+, CD38+, CD58+, CD79a+, CD19+, CD22+, CD33+, CD13+, CD7+, CD11b+	Morphology, MFC, Karyotyping, FISH, RT-PCR	Complex	*SET-NUP*,* WT1*,* FLT3-ITD*	CT	3	9	NR, died, +9	[10]
2021	B-ALL	M	22	-	-	-	Morphology, MFC, Karyotyping, FISH, RT-PCR	92,XXYY, i(17)(q10)×2[3]/46,XY[20]	*SET-NUP214*,*WT1*,*TCRδ*	CT	13	15	Relapsed, died	[10]
-	MPAL B/T	M	21	-	-	-	Morphology, MFC, Karyotyping, FISH, RT-PCR	Normal	-	HSCT	47	47	Relapsed, died, + 47	[11]
-	MPAL B/T	M	29	0.56	67.2	CD7, CD34, HLA-DR, CD10, CD19, CD33, CD117, cCD79a, cCD3	Morphology, MFC, Karyotyping, FISH, RT-PCR	46,XY, add(6), del(16)	-	CT+HSCT + CAR-T	7	53	CR, relapsed, died	[11]
-	B-ALL	M	35	-	-	-	Morphology, MFC, Karyotyping, FISH, RT-PCR	Normal	-	-	59	59	CR, alive,+59	[11]
-	MPAL B/T/M	M	45	-	-	-	Morphology, MFC, Karyotyping, FISH, RT-PCR	Normal	-	-	27	27	CR, alive,+27	[11]
2023	B-ALL	M	26	57.86	95.5	CD34,CD38,CD7,HLA-DR, CD58,CD19dim, cCD79a, CD13/CD33	Morphology, MFC, Karyotyping, FISH, RT-PCR, NGS	46,XY, del(11]	*JAK3*,*PHF6*,*JAK1*,*PTPN11*	CT+venetoclax+allo-HSCT	1	31	CR, relapsed, alive	present report

B-ALL, B-cell acute lymphoblastic leukemia; MPAL B/M, mixed phenotype acute leukemia B/myeloid; MPAL B/T, mixed phenotype acute leukemia B/T; MPAL B/M/T, mixed phenotype acute leukemia B/myeloid/T; HSCT, hematopoietic stem cell transplantation; CR: complete remission; CT, chemotherapy; CAR-T, chimeric antigen receptor T-cell immunotherapy; del, deletion; t,translocation; i,insertion; *FLT3-ITD*,FMS-like tyrosine kinase 3 internal tandem duplication;*SET-NUP*,suppress of variegation, enhancer of zeste, trithorax-nucleoporin;*WT1*,wilms tumor 1;*TCRδ*,T-cell receptor delta chain;*JAK3*,janus kinase 3;*PHF6*,plant homeodomain-like finger 6;*JAK1*,janus kinase 1;*PTPN11*, protein tyrosine phosphatase non-receptor type 11;RFS, relapse-free survival; OS, overall survival; WBC, white blood count; CD, cluster of differentiation; F,female; M,male; P, positive; N,negative; (+), positive; (-), negative

## Discussion

The *SET-NUP214* fusion gene (formerly designated as *SET-CAN*) arises from either a balanced t(9;9)(q34;q34) translocation or a cryptic del(9)(q34.11q34.13) deletion. While this genetic alteration is a well-characterized hallmark of T-ALL, its occurrence in the B-cell lineage (B-ALL) remains exceptionally rare. To our knowledge, this report constitutes the first documented case of relapsed/refractory *SET-NUP214*-positive B-ALL successfully salvaged with a venetoclax-based combination regimen.

*SET-NUP214*-positive leukemia is a clinically aggressive entity, typically exhibiting a distinct demographic predilection for young males (median age: 27.5–33 years) and consistent immunophenotypic aberrations—findings corroborated by both our index case and existing literature [12–14]. A significant diagnostic challenge lies in the fact that conventional cytogenetics often fails to detect this fusion; most cases present with either a normal or highly complex karyotype [15, 16]. Consequently, the diagnosis necessitates high-sensitivity modalities, such as FISH, targeted fusion gene screening, or transcriptome sequencing. In the present case, flow cytometry unequivocally established a B-lymphoid lineage with aberrant myeloid antigen expression (CD19⁺/dim, cCD79a⁺, CD34⁺, CD13⁺/33⁺), notably devoid of T-lineage commitment. However, molecular profiling underscores divergent mutational landscapes between lineages. While *SET-NUP214*-positive T-ALL is frequently associated with *NOTCH1/FBXW7* mutations, *PHF6* inactivation, and *JAK-STAT* pathway signaling aberrations (e.g., *JAK1/3*,* STAT5B*), the mutational signature in the B-cell context remains less defined, necessitating further genomic elucidation.

The incidence of the *SET-NUP214* fusion in acute leukemia is notably low. Utilizing multiplex-nested RT-PCR, Chen et al. identified a stark lineage disparity in the incidence of this fusion. Subgroup analysis revealed an incidence of 1.27% in T-ALL (ranking second) compared to a mere 0.29% in B-ALL (ranking ninth) [2]. This observation is further supported by recent large-scale genomic profiling of pediatric and adolescent/young adult (AYA) patients by Pölönen et al., which underscores the recurrent nature of the *SET::NUP214* fusion as a critical driver within this specific age demographic [17]. Recent studies have reported a broader incidence range in T-ALL, spanning from 0.78% to 10.3% [12, 18], while in the AML setting, Zhu et al. reported a frequency of 0.21% [13]. In a comprehensive cohort of 42 *SET-NUP214*-positive acute leukemia cases, T-ALL predominated (90.5%), with AUL (4.8%), AML (2.4%), and B-ALL (2.4%) constituting only a fraction of cases [7]. To date, documented B-ALL cases harboring the *SET-NUP214* rearrangement remain exceedingly scarce (*n* = 10). A hallmark of *SET-NUP214*-positive leukemia is the high prevalence of lineage infidelity or mixed-phenotype features. Although initial remission rates with conventional induction chemotherapy appear deceptively favorable (70.8–91.7%) [7, 12], universal corticosteroid resistance and an inherent propensity for rapid relapse pose formidable clinical challenges. The recurrent nature of *SET-NUP214* in B-lineage leukemia is further exemplified by the report from Nowak et al. [6], where cryptic 9q34 deletions were identified in adult patients with normal-karyotype B-ALL. The fact that these patients experienced rapid disease progression and limited survival with standard chemotherapy highlights the aggressive biology of this molecular subset. The same holds true for the *SET-NUP214* fusion in acute myeloid leukemia. Shen et al. [19] found that even after allogeneic stem cell transplantation, patients with this fusion continue to face a high risk of relapse, with estimated 1-year and 3-year leukemia-free survival of 69.3% and 38.5%, respectively. This aggressive disease biology was exemplified by our patient, who experienced early hematological relapse post-induction and remained refractory to subsequent conventional salvage regimens. While allo-HSCT significantly improves long-term outcomes in this cohort [7], achieving a deep second remission remains a critical and mandatory prerequisite for a successful bridge to transplantation in the relapsed/refractory setting.

The precise mechanisms by which *SET-NUP214* orchestrates leukemogenesis remain to be fully elucidated; however, current evidence suggests a multifactorial oncogenic program. The *SET* protein, acting as a histone chaperone, disrupts epigenetic homeostasis via the inhibition of protein phosphatase 2 A (PP2A). Concurrently, the FG-repeat domain derived from *NUP214* impairs nuclear pore integrity through the aberrant sequestration of *hCRM1/XPO1*, leading to nucleocytoplasmic transport defects and subsequent genomic instability [20, 21]. Although mouse models indicate that *SET-NUP214* can arrest hematopoietic differentiation across erythroid, megakaryocytic, and B-cell lineages, leukemic transformation appears to necessitate cooperating genetic hits [21]. The *SET-NUP214* fusion protein has been shown to recruit *MLL* complexes to the Homeobox A (*HOXA*) cluster, thereby sustaining a progenitor-like transcriptional program. This process is mediated through direct interaction with the *HOXA9/10* promoters, *DOT1L*-dependent epigenetic activation, and *XPO1*-mediated nuclear export dysregulation [22–24]. Crucially, *SET-NUP214*-driven *HOXA* overexpression creates a survival advantage for leukemic blasts [23, 25]; specifically, *HOXA9* has been identified as a direct upstream regulator of *BCL-2* in myeloid progenitors [26]. In our case, the robust *BCL-2* expression confirmed by immunohistochemistry aligns with this regulatory axis and likely underscores a key survival/resistance mechanism. Given that *HOXA/HOXB* overexpression serves as a potent predictor of venetoclax sensitivity in AML [27], and that *HOXA*-positive T-ALLs exhibit profound vulnerability to *BCL-2* inhibition [28], the rapid remission achieved in our patient suggests that venetoclax effectively exploits the dependency of *SET-NUP214*-positive B-ALL on the *HOXA/BCL-2* axis.

Venetoclax, a highly selective *BCL-2* inhibitor currently approved for the treatment of AML and chronic lymphocytic leukemia (CLL), has also emerged as a promising therapeutic option in ALL [29–33]. Beyond the present case, a limited number of reports have described the use of venetoclax in relapsed/refractory (R/R) *SET-NUP214*-positive acute leukemias. Shen et al. documented a patient with *SET-NUP214*-rearranged AML who experienced a molecular relapse post-transplantation; the patient successfully achieved a second remission and sustained survival following a combination of venetoclax, chemotherapy, and donor lymphocyte infusion (DLI) [19]. Similarly, Chen et al. described a case of chronic myeloid leukemia in myeloid blast crisis (CML-BC) co-harboring *BCR-ABL* and *SET-NUP214* mutations, where a triplet regimen of venetoclax, decitabine, and ponatinib facilitated a successful bridge to allo-HSCT [34]. Furthermore, in the T-cell lineage, Khanlar et al. demonstrated that venetoclax combined with decitabine effectively decimated the MRD burden in a patient with *SET-NUP214*-positive T-lymphoblastic leukemia/lymphoma [35]. Based on these cases, *SET-NUP214*-positive acute leukemia of different hematopoietic lineages demonstrates comparable sensitivity to venetoclax treatment. Nevertheless, the definitive efficacy of *BCL-2* inhibitors, such as venetoclax, in *SET-NUP214*-mutated acute leukemias remains to be confirmed by large-scale clinical trials.

In summary, *SET-NUP214*-positive B-ALL is an exceptionally rare entity characterized by chemoresistance and highly relapse rate, for which allo-HSCT remains crucial for improving prognosis. For patients with relapsed/refractory disease, achieving remission again prior to transplantation poses a significant challenge. This paper reports the first case of a patient with relapsed/refractory *SET-NUP214*-positive B-ALL who achieved complete remission following venetoclax combined with chemotherapy and attained long-term survival through allo-HSCT. The present case suggests that *BCL-2* inhibition may overcome the intrinsic resistance associated with the *SET-NUP214* fusion.

## Supplementary Information

Below is the link to the electronic supplementary material.

Supplementary Material 1

## Data Availability

No datasets were generated or analysed during the current study.

## References

[1] von Lindern M, Breems D, van Baal S, Adriaansen H, Grosveld G (1992) Characterization of the translocation breakpoint sequences of two DEK-CAN fusion genes present in t(6;9) acute myeloid leukemia and a SET-CAN fusion gene found in a case of acute undifferentiated leukemia. Genes Chromosomes Cancer 5(3):227–234 10.1002/gcc.2870050309 1384675

[2] Chen X, Wang F, Zhang Y, Wang M, Tian W, Teng W, Ma X, Guo L, Fang J, Zhang Y, Zhu P, Liu H (2018) Retrospective analysis of 36 fusion genes in 2479 Chinese patients of de novo acute lymphoblastic leukemia. Leuk Res 72:99–104 10.1016/j.leukres.2018.08.009 30125757

[3] Ben Abdelali R, Roggy A, Leguay T, Cieslak A, Renneville A, Touzart A, Banos A, Randriamalala E, Caillot D, Lioure B, Devidas A, Mossafa H, Preudhomme C, Ifrah N, Dombret H, Macintyre E, Asnafi V (2014) SET-NUP214 is a recurrent γδ lineage-specific fusion transcript associated with corticosteroid/chemotherapy resistance in adult T-ALL. Blood 123(12):1860–1863 10.1182/blood-2013-08-521518 24449214

[4] Kim J, Lee SG, Song J, Kim SJ, Rha SY, Lee KA, Park TS, Choi JR (2010) Molecular characterization of alternative SET-NUP214 fusion transcripts in a case of acute undifferentiated leukemia. Cancer Genet Cytogenet 201(2):73–80 10.1016/j.cancergencyto.2010.05.010 20682390

[5] Gorello P, Starza RL, Varasano E, Chiaretti S, Elia L, Pierini V, Barba G, Brandimarte L, Crescenzi B, Vitale A, Messina M, Grammatico S, Mancini M, Matteucci C, Bardi A, Guarini A, Martelli MF, R Foà, and, Mecucci C (2010) Combined interphase fluorescence in situ hybridization elucidates the genetic heterogeneity of T-cell acute lymphoblastic leukemia in adults. Haematologica 95(1):79–86 10.3324/haematol.2009.010413 PMC2805748 20065082

[6] Nowak NJ, Sait SN, Zeidan A, Deeb G, Gaile D, Liu S, Ford L, Wallace PK, Wang ES, Wetzler M (2010) Recurrent deletion of 9q34 in adult normal karyotype precursor B-cell acute lymphoblastic leukemia. Cancer Genet Cytogenet 199(1):15–20 10.1016/j.cancergencyto.2010.01.014 PMC2862995 20417863

[7] Zhu H, Zhao X, Qin Y, Lai Y, Jiang HAO (2016) B-cell acute lymphoblastic leukemia associated with SET-NUP214 rearrangement: A case report and review of the literature. Oncol Lett 11(4):2644–2650 10.3892/ol.2016.4260 PMC4812124 27073532

[8] Chen Y, Cen JN, Wang Q (2016) Study on mixed phenotype acute leukemia with SET-NUP214 fusion gene and myeloid sarcoma. Chin J Hemorheology 26(3):283–286

[9] Li MY, Lin ZH, Hu MM, Kang LQ, Wu XX, Chen QW, Kong X, Zhang J, Qiu HY, Wu DP (2020) Secondary donor-derived humanized CD19-modified CAR-T cells induce remission in relapsed/refractory mixed phenotype acute leukemia after allogeneic hematopoietic stem cell transplantation: a case report. Biomark Res 8:36 10.1186/s40364-020-00216-1 PMC7457525 32874588

[10] Chen SM, Song WJ, Qin YZ, Wang Z, Dang H, Shi Y, He Q, Jiang Q, Jiang H, Huang XJ, Lai YY (2021) Analysis of the clinical characteristics of 24 cases of hematological malignancies with SET-NUP214 fusion gene. Zhonghua Xue Ye Xue Za Zhi 42(6):459–465 10.3760/cma.j.issn.0253-2727.2021.06.004 PMC8295622 34384151

[11] Xia J, Zhao Y, Chen F, Miao M, Qiu HY, Ma X, Tang XW, Wang Y, Wu XJ, Fu ZZ, Wu DP, Chen SN (2023) Allogeneic hematopoietic stem cell transplantation in acute leukemia patients with the SET–NUP214 fusion gene: Efficacy and survival analysis. Chin J Intern Med 62(4):410–41 10.3760/cma.j.cn112138-20220411-00264 37032136

[12] Ben Abdelali R, Roggy A, Leguay T, Cieslak A, Renneville A, Touzart A, Banos A, Randriamalala E, Caillot D, Lioure B, Devidas A, Mossafa H, Preudhomme C, Ifrah N, Dombret H, Macintyre E, Asnafi V (2014) SET-NUP214 is a recurrent γδ lineage-specific fusion transcript associated with corticosteroid/chemotherapy resistance in adult T-ALL. Blood 123(12):1860–1863 10.1182/blood-2013-08-521518 24449214

[13] Geng Z, Zhang H, Wang D, Xiao Y, Wang N, Li C, Huang L, Zhou J (2012) Combination of cytogenetic analysis and molecular screening in patients with de novo acute myeloid leukemia. J Huazhong Univ Sci Technol [Medical Sciences] 32(4):501–510 10.1007/s11596-012-0087-6 22886961

[14] Yang Q, Qian H, Jin Z, Yu Z, Yu K, Zhang S, Jiang S (2020) SET-CAN fusion gene as poor prognosis predictor in adult T-cell acute lymphoblastic leukemia. Leuk Lymphoma 61(1):217–220 10.1080/10428194.2019.1660966 31502502

[15] Lee SG, Park TS, Cho SY, Lim G, Park GJ, Oh SH, Cho EH, Chong SY, Huh JY (2011) T-cell acute lymphoblastic leukemia associated with complex karyotype and SET-NUP214 rearrangement: a case study and review of the literature. Ann Clin Lab Sci 41(3):267–272 22075511

[16] Chen SM, Song WJ, Qin YZ, Wang Z, Dang H, Shi Y, He Q, Jiang Q, Jiang H, Huang J, Lai YY(2021) Analysis of the clinical characteristics of 24 cases of hematological malignancies with SET-NUP214 fusion gene. Zhonghua Xue Ye Xue Za Zhi(in chinese) 42(6):459–46 10.3760/cma.j.issn.0253-2727.2021.06.004 PMC8295622 34384151

[17] Pölönen P, Giacomo DD, Seffernick AE, Elsayed A, Kimura S, Benini F, Montefiori LE, Wood BL, Xu J, Chen C, Cheng Z, Newman H, Myers J, Iacobucci I, Li E, Sussman J, Hedges D, Hui Y, Diorio C, Uppuluri L, Frank D, Fan Y, Chang Y, Meshinchi S, Ries R, Shraim R, Li A, Bernt KM, Devidas M, Winter SS, Dunsmore KP, Inaba H, Carroll WL, Ramirez NC, Phillips AH, Kriwacki RW, Yang JJ, Vincent TL, Zhao Y, Ghate PS, Wang J, Reilly C, Zhou X, Sanders MA, Takita J, Kato M, Takasugi N, Chang BH, Press RD, Loh M, Rampersaud E, Raetz E, Hunger SP, Tan K, Chang TC, Wu G, Pounds SB (2024) CG Mullighan, and DT Teachey, *The genomic basis of childhood T-lineage acute lymphoblastic leukaemia*. Nature 632(8027):1082–1091 10.1038/s41586-024-07807-0 PMC11611067 39143224

[18] Dai HP, Wang Q, Wu LL, Ping NN, Wu CX, Xie JD, Pan JL, Xue YQ, Wu DP, Chen SN (2012) Expression of SET-NUP214 fusion gene in patients with T-cell acute lymphoblastic leukemia and its clinical significance. Zhongguo Shi Yan Xue Ye Xue Za Zhi 20(5):1047–1051 23114116

[19] Shen Y, Yang D, Zhang R, Chen X, Ma Q, Wei J, Zhai W, Pang A, He Y, Jiang E, Feng S (2023) The outcome of acute leukemia patients with SET-NUP214 fusion after allogeneic stem cell transplantation. Front Oncol 13:1256043 10.3389/fonc.2023.1256043 PMC10611484 37901324

[20] Song J, Li H, Fan S (2023) SET-CAN/NUP214 fusion gene in leukemia: general features and clinical advances. Front Oncol 13:1269531 10.3389/fonc.2023.1269531 PMC10613893 37909026

[21] Saito S, Nouno K, Shimizu R, Yamamoto M, Nagata K (2007) Impairment of erythroid and megakaryocytic differentiation by a Leukemia-Associated and t(9;9)‐derived fusion gene product, SET/TAF‐Iβ‐CAN/Nup214. J Cell Physiol 214(2):322–333 10.1002/jcp.21199 17620317

[22] Zhou M-H, Yang Q-M (2014) NUP214 fusion genes in acute leukemia (Review). Oncol Lett 8(3):959–962 10.3892/ol.2014.2263 PMC4114590 25120641

[23] Oka M, Mura S, Otani M, Miyamoto Y, Nogami J, Maehara K, Harada A, Tachibana T, Yoneda Y, Ohkawa Y (2019) Chromatin-bound CRM1 recruits SET-Nup214 and NPM1c onto HOX clusters causing aberrant HOX expression in leukemia cells. eLife 8:e46667 10.7554/eLife.46667 PMC6874418 31755865

[24] Mendes A, Fahrenkrog B (2019) NUP214 in leukemia: it’s more than transport. Cells 8(1):76 10.3390/cells8010076 PMC6356203 30669574

[25] Van Vlierberghe P, van Grotel M, Tchinda J, Lee C, Beverloo HB, van der Spek PJ, Stubbs A, Cools J, Nagata K, Fornerod M, Buijs-Gladdines J, Horstmann M, van Wering ER, Soulier J, Pieters R, Meijerink JPP (2008) The recurrent SET-NUP214 fusion as a new HOXA activation mechanism in pediatric T-cell acute lymphoblastic leukemia. Blood 111(9):4668–4680 10.1182/blood-2007-09-111872 PMC2343598 18299449

[26] Brumatti G, Salmanidis M, Kok CH, Bilardi RA, Sandow JJ, Silke N, Mason K, Visser J, Jabbour AM, Glaser SP, Okamoto T, Bouillet P (2013) RJ D’Andrea, and PG Ekert, *HoxA9 regulated Bcl-2 expression mediates survival of myeloid progenitors and the severity of HoxA9-dependent leukemia*. Oncotarget 4(11):1933–1947 10.18632/oncotarget.1306 PMC3875760 24177192

[27] Kontro M, Kumar A, Majumder MM, Eldfors S, Parsons A, Pemovska T, Saarela J, Yadav B, Malani D, Fløisand Y, Höglund M, Remes K, Gjertsen BT, Kallioniemi O, Wennerberg K, Heckman CA, Porkka K (2016) HOX gene expression predicts response to BCL-2 inhibition in acute myeloid leukemia. Leukemia 31(2):301–309 10.1038/leu.2016.222 27499136

[28] Peirs S, Matthijssens F, Goossens S, Van de Walle I, Ruggero K, de Bock CE, Degryse S, Canté-Barrett K, Briot D, Clappier E, Lammens T, De Moerloose B, Benoit Y, Poppe B, Meijerink JP, Cools J, Soulier J, Rabbitts TH, Taghon T, Speleman F, Van Vlierberghe P (2014) ABT-199 mediated inhibition of BCL-2 as a novel therapeutic strategy in T-cell acute lymphoblastic leukemia. Blood 124(25):3738–3747 10.1182/blood-2014-05-574566 25301704

[29] Pullarkat VA, Lacayo NJ, Jabbour E, Rubnitz JE, Bajel A, Laetsch TW, Leonard J, Colace SI, Khaw SL, Fleming SA, Mattison RJ, Norris R, Opferman JT, Roberts KG, Zhao Y, Qu C, Badawi M, Schmidt M, Tong B, Pesko JC, Sun Y, Ross JA, Vishwamitra D, Rosenwinkel L, Kim SY, Jacobson A, Mullighan CG, Alexander TB, Stock W (2021) Venetoclax and Navitoclax in Combination with Chemotherapy in Patients with Relapsed or Refractory Acute Lymphoblastic Leukemia and Lymphoblastic Lymphoma. Cancer Discov 11(6):1440–1453 10.1158/2159-8290.CD-20-1465 PMC9533326 33593877

[30] Gibson A, Trabal A, McCall D, Khazal S, Toepfer L, Bell DH, Roth M, Mahadeo KM, Nunez C, Short NJ, DiNardo C, Konopleva M, Issa GC, Ravandi F, Jain N, Borthakur G, Kantarjian HM, Jabbour E, Cuglievan B (2022) Venetoclax for children and adolescents with acute lymphoblastic leukemia and lymphoblastic lymphoma. Cancers (Basel) 14(1):150 10.3390/cancers14010150 PMC8750927 35008312

[31] Goethe University Frankfurt (2021) An open label, phase I/II study of Venetoclax in addition to Blinatumomab immunotherapy in adult patients with relapsed/refractory B cell precursor acute lymphoblastic leukemia (BCP-ALL). ClinicalTrials.gov identifier NCT05182385; EudraCT number 2021-001384-25

[32] Sun H, Zhu Y, Li J, Zhao L, Yang G, Yan Z, Zhang S (2024) PICALM::MLLT10 may indicate a new subgroup of acute leukemias with miscellaneous immunophenotype and poor initial treatment response but showing sensitivity to venetoclax. EJHaem 5(3):565–572 10.1002/jha2.922 PMC11182389 38895061

[33] Zhang L, Zhang Z, Lu A, Jia Y, Huang L, Zhang L, Zeng H (2026) Venetoclax-based regimen in refractory or relapsed pediatric acute lymphoblastic leukemia. Acta Haematol 149(3):280–286 10.1159/000547080 40628245

[34] Chen Y, Wang Q, Cen J, Xu C, Tao TT, Xie J, Shen W, Gong Y, Pan J, Yao L (2023) Blast phase of chronic myeloid leukemia with concurrent BCR::ABL1 and SET::NUP214: A report of two cases. Mol Carcinog 62(2):117–121 10.1002/mc.23480 36321418

[35] Khanlari M, Wang W, Mead PE, Key L, Liu P, Thakral B, Blackburn PR, Klco JM, Ribeiro R, Sarvode S (2025) Pediatric T-Lymphoblastic Leukemia With Aberrant B-Cell Marker Expression: A Potential Role for Targeted Therapy. EJHaem 6(6):e70191 10.1002/jha2.70191 PMC12648628 41311531

