# Supplementary material for: Successful treatment of B-cell acute lymphoblastic leukemia with SET-NUP214 rearrangement using venetoclax: A first case report and literature review
Source: Ann Hematol. 2026 Jul 28;105(11):481. doi: 10.1007/s00277-026-07190-y (PMC13696570; doi:10.1007/s00277-026-07190-y)
Supplement: Supplementary file 1 [file 277_2026_7190_MOESM1_ESM.docx]

1. Supplementary Material 1: the patient's original flow cytometry.

Multiparameter Flow Cytometry (MFC) Immunophenotyping Report

Specimen: Bone Marrow (BM)

Blast Population: 94.3% of total nucleated cells (Gated as P4).

Immunophenotype:

Positive: CD34, CD38, HLA-DR, CD7, CD58.

Partial: CD19dim, cCD79a, CD13+33.

Negative: cCD22, CD10, CD20, CD9, CD117, CD81, CD123, nTdT, CD66c, cIgM, CD3, CD5, CD138, cCD3, cMPO, CD15, CD2, CD1a, CD4, CD8, TCRab, CD56.

Conclusion: The immunophenotype is consistent with B-cell Acute Lymphoblastic Leukemia (Pro-B-ALL).

Supplementary Figure A and B. Original Flow Cytometry Diagnostic Findings:

Figure A. Immunophenotypic Population Summary (Statistical Data). This table summarizes the distribution of cell populations analyzed by multiparameter flow cytometry (MFC). A distinct cluster of Blasts (labeled as P4) was identified, accounting for 94.3% of the parent cell population and 85.0% of the total events. L, Lymphocytes; P, Granulocytes; P4/Blasts, Primitive cells (Blasts); M, Monocytes (if applicable); N, Nucleated Red Blood Cells.

Figure B. Multiparameter Flow Cytometric Scatter Plots (Gating and Phenotyping) Representative dot plots showing the gating strategy and immunophenotype of the bone marrow aspirate. Cells were initially gated using CD45 vs. Side Scatter (SSC) to identify the blast population (red cluster).

2. Supplementary Material 2: Bone Marrow Pathology Report.

1). Macroscopic Description:

Specimen: One bone marrow core biopsy, measuring approximately 1.5 cm in length.

2). Microscopic Findings (Pathological Description)

- Cellularity: The bone marrow is markedly hypercellular (approaching 100% cellularity).
- Hematopoietic Lineages: Granulocytic & Erythroid Series: Both series are significantly reduced. Megakaryocytes: Occasionally observed.
- Blast Infiltration: There is a diffuse and massive infiltration of primitive cells (blasts). These cells are medium-to-large in size, with scanty cytoplasm, fine chromatin, and inconspicuous nucleoli.
- Stromal Changes: No obvious evidence of fibrosis or necrosis is noted within the examined sections.

3). Histochemical and Immunohistochemical Staining

I. Histochemical Staining:

- Reticulin Staining: Positive (+), MF grade 1.
- Iron Staining: Positive (+).

II. Immunohistochemical Staining (IHC):

- Positive: CD19, CD10, CD34.
- Scattered Positive: TdT.
- Few Cells Positive: CD3, MPO.

4). Pathological Diagnosis: Consistent with B-lymphoblastic leukemia/lymphoma (B-ALL).

Figure C. Bone Marrow Biopsy Images.

3. Supplementary Material 3: Detailed genomic profile of the patient at initial diagnosis.

| Gene | Variant (Protein level) | Variant (cDNA level) | Variant Type | VAF (DNA, %) | VAF (RNA, %) |
| --- | --- | --- | --- | --- | --- |
| *JAK3* | p.M511I | c.1533G>C | Missense (Exon 11) | 48.56% | 37.14% |
| *PHF6* | p.D264Vfs*14 | c.791_800delinsTCGGTG | Frameshift (Exon 8) | 94.79% | 100.00% |
| *JAK1* | p.E897K | c.2689G>A | Missense (Exon 20) | 20.48% | 16.08% |
| *JAK3* | p.V674F | c.2020G>T | Missense (Exon 15) | 24.03% | 23.93% |
| *PTPN11* | p.A465G | c.1394C>G | Missense (Exon 12) | 48.35% | 46.67% |
| *SET::NUP214* | Fusion | SET:exon7~NUP214:exon18 | Gene Fusion | - | 4.38% |
